# Winning the Battle after Three Years of Suffering: A Case of a Refractory Pyoderma Gangrenosum Treatment Challenge

**DOI:** 10.1155/2021/8869914

**Published:** 2021-03-13

**Authors:** Isra Ibrahim, Hammam Shereef, Ahmed Hashim, Heba Habbal, Raai Mahmood, Mohamed A. Mohamed

**Affiliations:** ^1^Department of Internal Medicine, Lutheran Hospital, Fort Wayne, IN, USA; ^2^Department of Internal Medicine, Beaumont Hospital-Dearborn, Dearborn, MI, USA; ^3^Division of Rheumatology, Department of Internal Medicine, Reid Healthcare System, Indianapolis, IN, USA

## Abstract

Pyoderma gangrenosum is an uncommon inflammatory disorder characterized by neutrophilic infiltration of the skin. It can present as skin papules or pustules that progress into painful ulcers. 30–40% of the cases are associated with other systemic diseases such as inflammatory bowel diseases, rheumatoid arthritis, and proliferative hematological disorders. Uniquely, this condition has been associated with systemic lupus erythematosus (SLE). The rarity of this disorder poses a diagnostic and therapeutic challenge. We present a case of a 55-year-old female with a history of SLE and chronic right leg ulcer, presented with increased pain from the ulcer associated with a mild flare of her cutaneous lupus; examination revealed circumferential skin ulcer measuring about 25 cm extending around the right leg above the ankle with prominent fibrinous material and surrounding erythema. Blood work showed elevated WBC with neutrophilic predominance. Serology revealed a positive ANA, elevated RNP, smith, and SSA/Ro antibodies with normal anti-CCP level. Skin biopsy was taken, and it showed a diffuse neutrophilic and lymphocytic infiltrate consistent with the diagnosis of pyoderma gangrenosum. The patient was then treated with topical and systemic steroids and sequentially with dapsone, methotrexate, mycophenolate, and cyclosporine for over a two-year period but failed to show any improvement. Therefore, a trial of intravenous immunoglobulin (IVIG) therapy was attempted and produced a dramatic response after two-month infusions characterized by shrinking in the size of the ulcer and resolving pain. We believe that refractory PG poses a therapeutic challenge, and despite a lack of specific guidelines, IVIG can be attempted if initial suppressive treatment fails to show signs of improvement.

## 1. Introduction

Pyoderma gangrenosum (PG) is an uncommon inflammatory neutrophilic disorder (ND) characterized by neutrophilic infiltration of the skin with an estimated incidence of 3–10 cases per million/year person [1]. PG can present as skin papules or pustules that progress into painful ulcers, 30–40% of the cases are associated with other systemic diseases such as inflammatory bowel diseases (IBD), rheumatoid arthritis, hemoproliferative and hematological disorders, PAPA (pyogenic sterile arthritis, pyoderma gangrenosum, Acne) syndrome, and PASH (pyoderma gangrenosum, acne, suppurative hidradenitis) syndrome [2–4]. PG predominately affects women, with an average age of onset between 40 and 60 years [5, 6]. The rarity of this disorder poses a challenge when it comes to diagnosis and treatment.

### 1.1. Case Presentation

**Figure 1:**
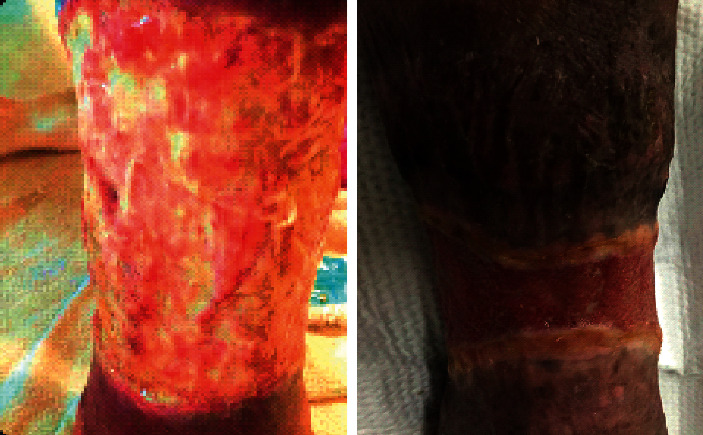
Skin ulcer with prominent fibrinous material and surrounding erythema.

A 55-year-old woman with a past medical history of SLE diagnosed based on the American College of Rheumatology (ACR) with the presence of typical cutaneous lesions and immunochemical tests presents with chronic right leg ulcer that is unresponsive to treatment; she had increased pain and discharge from the ulcer, and during the same time, she had a mild flare of her cutaneous lupus lesions with new erythematous skin rash involving forehead, cheeks, extensor surface of both elbows, and also worsening Raynaud with multiple fingertip ulcers. Over a period of few months prior to our evaluation, the patient had multiple admissions for presumed infections of chronic right leg ulcer, so she was treated with a short course of antibiotics in addition to wound care. Physical examination showed a malar rash on her face and a circumferential skin ulcer measuring about 25 cm width, extending right lower leg above the ankle with prominent fibrinous material and surrounding erythema (Figure 1).

### 1.2. Differential Diagnosis

At this point, the differential diagnosis included vascular insufficiency, infectious process, and drug-induced skin necrosis.

### 1.3. Investigations

**Figure 2:**
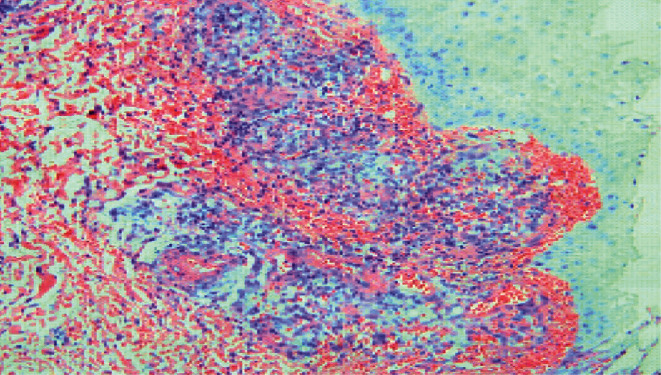
Histopathology exam showing diffuse neutrophilic and lymphocytic infiltrate.

Initial blood work showed elevated WBC with neutrophilic predominance. Serological testing revealed positive ANA, positive RNP antibody, positive anti-smith antibody, positive SSA/Ro antibody, and normal SSB/La and anti-CCP antibodies, wound cultures were obtained, and they were unrevealing. Venous Doppler and ankle-brachial index of lower limbs were obtained and were negative for venous and arterial insufficiency. Due to the chronicity of the ulcers and lack of response to conventional medications, skin biopsy of the ulcer margin was taken, and it showed a diffuse neutrophilic and lymphocytic infiltrate which was consistent with the diagnosis of pyoderma gangrenosum (Figure 2).

### 1.4. Treatment

The patient was initially treated with topical and systemic steroids and subsequently with dapsone, methotrexate, mycophenolate, and cyclosporine for over a two-year period, but she failed to show any signs of improvement; therefore, a trial of intravenous immunoglobulin therapy was attempted, and it produced a dramatic response after two-month infusions characterized by shrinking in the size of the ulcer with granulation tissue formation and resolving pain.

## 2. Discussion

Pyoderma gangrenosum presents initially as a papule, vesicle, or nodule that subsequently expands and breaks down to form a rapidly progressive painful ulcer which is a classic presentation of PG. Systemic diseases known to be associated with PG most commonly include IBD, autoimmune diseases such as RA, autoinflammatory syndromes such as PAPA and PASH syndromes, and hematologic malignancy (IgA monoclonal gammopathy, chronic myelogenous leukemia, and myelofibrosis). Although SLE has not been classically linked to PG, several case reports and case series in the literature found some relevant association between those two entities, and in some reports, PG was found to herald the onset of SLE [7–9]. Pathogenesis of PG is not fully understood, but newer theories suggest that abnormalities of neutrophil function, genetic variations, and dysregulation of the innate immune system are responsible for the disease [10, 11].

Updated diagnostic criteria for PG yield a sensitivity of 86% and a specificity of 90% when one major and four out of eight minor criteria are met for the diagnosis of PG as follows.

Major criterion (one) is as follows: biopsy of ulcer edge demonstrating neutrophilic infiltrate.

Minor criteria (eight) are as follows: (1) exclusion of infection; (2) pathergy; (3) history of inflammatory bowel diseases or inflammatory arthritides; (4) history of papule, pustule, or vesicle ulcerating within four days of appearing; (5) peripheral erythema, undermining border, and tenderness at ulceration site; (6) multiple ulcerations, at least 1 on an anterior lower leg; (7) cribriform or “wrinkled paper” scars at healed ulcer sites; and (8) decreased ulcer size within one month of initiating immunosuppressive medication [12].

Treatment of PG depends on the severity of the disease, and mild disease requires local measures only like topical high potency steroids or topical tacrolimus with proper wound care to promote healing. If local therapy fails, then systemic treatment with dapsone or minocycline is indicated. For patients with extensive disease, systemic corticosteroids, cyclosporine, infliximab, methotrexate, and azathioprine have been shown to be beneficial; however, refractory disease treatment options are limited. Intravenous immunoglobulins (IVIGs) had been tried before for such cases and showed promising results with complete response in 53% of the cases, yet the long-term benefit is not adequately determined [13]. The utilization of alkylating agents like cyclophosphamide is limited due to potential side effects and marrow suppression. Our patient fulfilled the updated criteria for the diagnosis of PG by virtue of proven skin biopsy and four of the minor criteria (tenderness at the ulceration site, lack of infection, good response to therapy, and wrinkled skin after treatment). She failed to respond to local and systemic steroids and upon escalating treatment to different immune-suppressive medications including dapsone cyclophosphamide, methotrexate, and mycophenolate, her pain and size of the ulcer did not change; therefore, we attempted a trial of intravenous immunoglobulin, and remarkably over a period of two months, she showed dramatic improvement with a decrease in the size of the ulcer and significant improvement in her pain.

## 3. Conclusion

Biopsy is especially important in the workup of chronic nonhealing skin ulcers to help ruling out malignancy and establishing a diagnosis. PG should be included in the differential diagnosis of chronic skin lesions especially in the presence of systemic disease. Refractory pyoderma gangrenosum may pose a therapeutic challenge and despite lack of specific guidelines, IVIG can be attempted if initial immunosuppressive treatment fails to show clinical improvement.
